# On-surface synthesis of nonbenzenoid nanographenes through skeletal rearrangement reactions on Au(111)

**DOI:** 10.1080/14686996.2026.2619342

**Published:** 2026-01-23

**Authors:** Kewei Sun, Xiushang Xu, Atsushi Ishikawa, Akimitsu Narita, Shigeki Kawai

**Affiliations:** a International Center for Young Scientists, National Institute for Materials Science, Tsukuba, Japan; b Center for Basic Research on Materials, National Institute for Materials Science, Tsukuba, Japan; c Organic and Carbon Nanomaterials Unit, Okinawa Institute of Science and Technology Graduate University, Okinawa, Japan; d Department of Transdisciplinary Science and Engineering, School of Environment and Society, Institute of Science Tokyo, Meguro-Ku, Japan; e Graduate School of Pure and Applied Sciences, University of Tsukuba, Tsukuba, Japan

**Keywords:** On-surface synthesis, scanning tunneling microscopy, nanographene, spin

## Abstract

Skeletal rearrangement reaction is a class of important chemical reactions to synthesize various constitutional isomers from a given precursor molecule. This approach allows the synthesis of diverse nanographenes incorporating five- and seven-membered rings, thereby tuning their chemical and physical properties. Here, we present formation of five types of closed-shell and two types of open-shell nanographenes on Au(111) via carbon rearrangements of 7-(2,6-dimethylphenyl)-12-[10-(2,6-dimethylphenyl)anthracen-9-yl]tetraphene. The structural, electronic, and magnetic properties of various products composed of pentagonal or pentagonal/heptagonal rings were in-detail investigated with a combination of bond-resolved scanning tunneling microscopy/scanning tunneling spectroscopy at 4.3 K and density functional theory calculations. We found that both zigzag edges and fused pentagonal rings significantly affect the band gap and the spin polarization. This discovery could facilitate the on-surface synthesis of intriguing carbon nanostructures through skeletal rearrangement.

## IMPACT STATEMENT

We present on-surface synthesis of various nonbenzenoid nanographenes, including five closed-shell and two open-shell structures, through skeletal rearrangement reaction using a precursor molecule featuring an out-of-plane benzene ring.

## GRAPHICAL ABSTRACT

**Figure:**
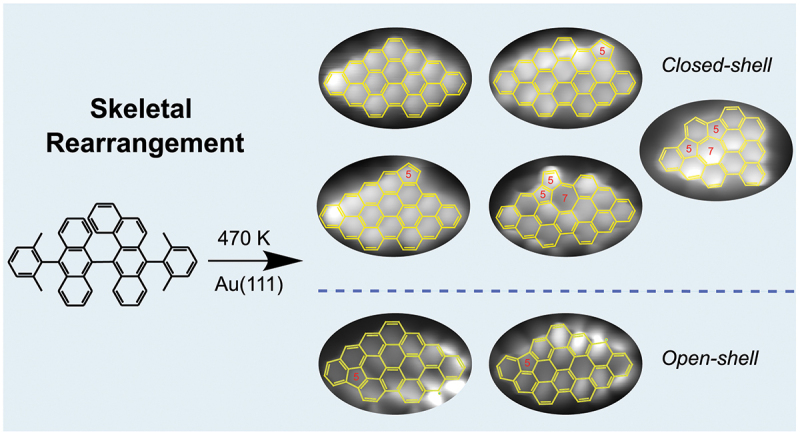

## 1. Introduction

Nanographenes (NGs) can serve as the core building blocks for carbon-based nanoelectronics, as their electronic, optical and magnetic properties can be tuned through edge topologies and molecular sizes [1,2]. Incorporation of nonbenzenoid rings into NGs can further modulate their chemical and physical properties. Rearrangement reaction is a class of organic reactions in synthetic chemistry, in which migration of atoms and/or groups in molecules results in constitutional isomerization. It is well known that Wagner – Meerwein rearrangement [3,4] and Beckmann rearrangement [5] as well as Pericyclic reaction [6] have been used to induce the isomerization in solution-based chemistry. For instance, pericyclic reactions often entail the intramolecular conversion between π bonds and σ bonds [7]. In this context, skeletal rearrangements, involving the reorganization of carbon atoms, provide an effective way to introduce nonbenzenoid rings into NGs [8–13].

On-surface synthesis became an important strategy to obtain low-dimensional carbon materials [14,15]. In the reaction, precursor molecules deposited on surfaces under ultra-high vacuum conditions are activated by metal catalysis, injected electrons and irradiated light, and subsequently conjugated with each other. Unlike molecular self-assembly [16,17], on-surface synthesis relies on covalent bonding between precursor molecules. Thus, their structures give a decisive role in the products. Since the electronic and magnetic properties of such nanocarbon materials can be tuned by the sizes and shapes as well as edge structures, it is of central importance to increase variety of the structures. To this end, several on-surface reactions have been developed, such as Ullmann-type coupling [18,19], Glaser-type coupling [20–22], Sonogashira-type cross-coupling [23–25] and dehydrogenation [26–28]. So far, various NGs [28–31], graphene nanoribbons (GNRs) [26,32–39] and covalent organic frameworks (COFs) [40–43] have been synthesized. The on-surface rearrangement reaction is also a powerful strategy for further modification of the structures [44–55]. However, the synthesis of multiple nonbenzenoid NGs with diverse properties from a single precursor through this approach remains scarce, and it is still unclear how to effectively trigger such rearrangements through appropriate precursor design.

Here, we report cyclodehydrogenation of 7-(2,6-dimethylphenyl)-12-[10-(2,6-dimethylphenyl)anthracen-9-yl]tetraphene (**1**) on Au(111), which underwent unique skeletal rearrangements to yield seven types of NGs. Importantly, the rearrangement is initiated by the spatial overlap of carbon atoms in the three-dimensional molecular configuration. Bond-resolved scanning tunneling microscopy (STM) shows the detailed structures of the products, in which penta- and hexa- as well as heptagonal rings are embedded at different sites. Their electronic properties were systematically investigated by scanning tunneling spectroscopy (STS) at 4.3 K. Among them, the presence of Kondo resonance at the Fermi level indicates that two types of NGs have open-shell characters, which is further confirmed by density functional theory (DFT) calculations. This method may provide guidance for the synthesis of additional nonbenzenoid NGs with tailored electronic and magnetic properties.

## 2. Experimental details

All the surface experiments were performed in a low temperature (STM) system (home-made) at 4.3 K under ultrahigh vacuum (<1 × 10^−10^ mbar). A clean single crystal Au(111) surface was prepared through cyclic sputtering (Ar^+^, 10 min) and annealing (720 K, 15 min). The temperature of the sample was measured by a thermocouple and a pyrometer. Molecules 7-(2,6-dimethylphenyl)-12-(10-(2,6-dimethylphenyl)anthracen-9-yl)tetraphene (**1**) were deposited from a Knudsen cell. A STM tip was made from a chemically etched tungsten wire. For constant-height d*I*/d*V* imaging, the tip apex was terminated by a carbon monoxide (CO) molecule picked up from the surface. The bias voltage was set close to zero voltage. The modulation amplitude of the bias voltage was 10 mV_ac_ and 0.3 mV_ac_ with the frequency was 510 Hz for STS measurement. Before the temperature dependent d*I*/d*V* measurements, the liquid helium in the cryostat was fully evaporated. Thus, the sample temperature gradually increased. The non-linear thermal drifts in the X, Y, and Z directions were thoroughly corrected by atom-tracking function before each d*I*/d*V* spectroscopy measurement. A similar protocol can be found in Ref [56].

To synthesis precursor, all reactions working with air- or moisture-sensitive compounds were carried out under argon atmosphere using standard Schlenk line techniques. All starting materials, reagents, and solvents were purchased from commercial sources and used as received unless otherwise noted. 7-Bromo-12-(10-bromoanthracen-9-yl)tetraphene (**2**) was prepared according to a previously reported procedure [57]. Anhydrous toluene was purified by a solvent purification system (GlassContour) prior to use. Thin-layer chromatography (TLC) was done on silica gel coated aluminum sheets with F254 indicator and column chromatography separation was performed with silica gel (particle size 0.063–0.200 mm). Nuclear Magnetic Resonance (NMR) spectra were recorded in CDCl_3_ using Bruker DPX 500 MHz NMR spectrometers. Chemical shifts (*δ*) were expressed in ppm relative to the residual of solvents (CDCl_3_, ^1^H: 7.26 ppm, ^13^C: 76.00 ppm). Coupling constants (*J*) were recorded in Hertz. Abbreviations: s = singlet, d = doublet, *t* = triplet, *m* = multiplet. High-resolution mass spectra (HRMS) were recorded on a Bruker ultrafleXtreme spectrometer by matrix-assisted laser decomposition/ionization (MALDI).

The density functional theory (DFT) calculations were performed to identify the electronic and magnetic properties of the π conjugated system adsorbed on the Au surface. Adsorbate molecules used in this study were **2**, **3**, and **6** (in Figure 2) for the density of states (DOS) calculation. For magnetic property calculation, **7**, **8** (in Figure 4) and their extended systems (**9** and **10** in Figure S14) were used. These were adsorbed on the Au surface to build the slab model. The Au surface was constructed from the face-centered cubic Au bulk structure, where an experimental lattice constant of 4.078 Å was used [58]. From this bulk structure, the 8 × 8 × 3 supercell was made to form the Au(111) surface, which contains 192 Au atoms. The bottom two Au layers were fixed during the calculations, while other parts were fully relaxed. Prior to the geometry optimization, the molecular dynamics (MD) calculations were performed to sufficiently explore the configuration space. The MD simulation was done for 0.5 ps, with 1.0 fs taken as the timestep. The canonical (*NVT*) ensemble with *T* = 200 K was used, where the Berendsen thermostat was used to control the temperature. After the MD calculation, the geometry optimization was carried out. For the electronic structure calculation, the projector augmented-wave (PAW) method was used to express the core and inner electrons, while plane-waves were used to express the valence electrons [59]. As the exchange-correlation functional, the restored regularized strongly constrained (R^2^SCAN) functional [60] was used throughout. The plane wave energy cutoff was set to 400 eV, and the reciprocal space integration was done with k-points generated according to the Monkhorst–Pack scheme. For MD calculation, single k-point at gamma was used, and other calculations were done with 3 × 3 × 1 k-points mesh. To alleviate the artificial interaction between slabs, a vacuum layer of 13 Å thickness was placed between slabs. The thresholds for the electronic structure calculation and geometry optimization were set to 1.0 × 10^−5^ eV in energy and 3.0 × 10^−2^ eV/Å in force. Spin polarization was considered throughout. For the smearing of the electron occupation, the 1^st^ order Methfessel-Paxton scheme was used for MD and geometry optimization, and the tetrahedron method was used for the DOS and spin density calculation. The partial charge densities in Figure 3 for highest occupied molecular orbital (HOMO) and lowest unoccupied molecular orbital (LUMO) was made by taking the energy interval of 0.2 eV around the HOMO and LUMO, i.e. from HOMO −0.1 eV to HOMO +0.1 eV. The charge density plot in Figure 3 corresponds to the isosurface of 0.001–0.003. The spin density plot of Figure 5 was made with the isosurface of 0.005. All the calculations were done with Vienna ab initio simulation package (VASP) version 6.4 [61,62]. The visualization was done with the visualization for electronic and structural analysis (VESTA) software [63].

## 3. Results and discussions

**Figure 1.**
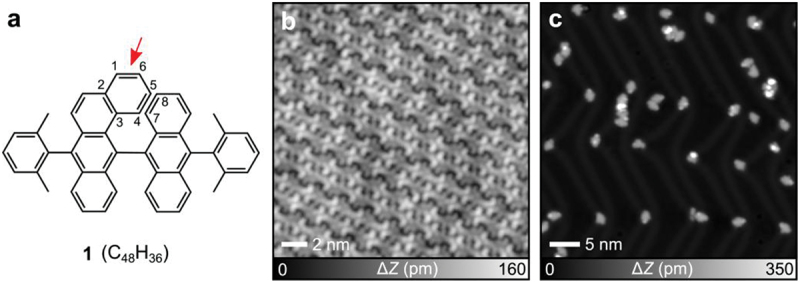
On-surface molecular rearrangement of **1** on Au(111). (a) Chemical structure of **1**. (b) STM topography of as-deposited **1** on Au(111). (c) STM topography of the sample after annealing at 470 K for 10 min. Measurement parameters: sample bias voltage *V* = 200 mV and tunneling current *I* = 5 pA in (b). *V* = 200 mV and *I* = 2 pA in (c).

To investigate skeletal rearrangement within single molecules, we employed molecule **1** (Figure 1a) as a precursor. The steric hindrance between C4-C5 and C7-C8 moieties induces the out-of-plane benzene ring as indicated by an arrow. We propose that this spatial overlap of carbon atoms facilitates the rearrangement reaction (Figure S1), leading to the formation and incorporation of nonbenzenoid moieties. The reaction in this study is based on on-surface cyclodehydrogenative planarization, which involves cleavage of the out-of-plane benzene ring and subsequent intramolecular rearrangement. As-deposited **1** (C_48_H_36_) on Au (111) at room temperature formed corrugated self-assemblies (Figure 1b), in which the brightest contrast corresponds to the out-of-plane benzene ring (Figure S2). Here, Au(111) was chosen because its relatively mild catalytic activity does not readily cleave the molecular C–C skeleton, unlike more reactive substrates such as Pt. Subsequently, cyclodehydrogenation was induced by annealing the sample at 470 K. Most molecules became planar (Figure S3) and adsorbed at the elbow sites of the herringbone reconstructed surface (Figure 1c). Annealing the sample at 520 K induced random fusion of these isolated molecules (Figure S4).

**Figure 2.**
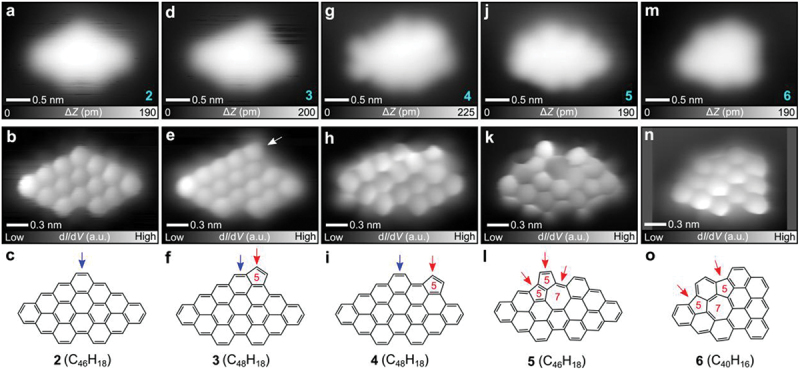
On-surface synthesis of closed-shell NGs. (a, d, g, j, m) close-up STM topographies of **2**, **3**, **4**, **5** and **6**, respectively. (b, e, h, k, n) corresponding bond-resolved images of **2**, **3**, **4, 5** and **6**, respectively. (c, f, i, l, o) chemical structures of **2**, **3**, **4**, **5** and **6**, respectively. Measurement parameters: *V* = 200 mV and *I* = 5 pA in (a, d, g, j). *V* = 200 mV and *I* = 10 pA in m. *V* = 1 mV, *V*_ac_ = 10 mV in (b, e, h, k, n).

**Scheme 1.**
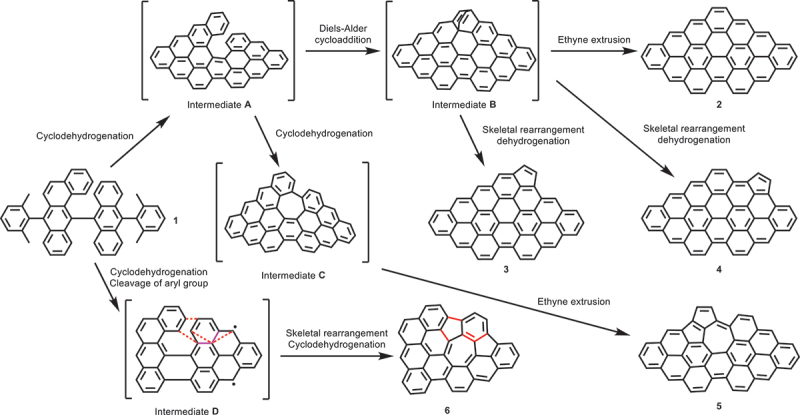
Proposed intermediate structures leading to NGs **2**–**6** during the on-surface reactions of precursor **1** involving skeletal rearrangements. Red dotted lines and pink solid lines in intermediate **D** indicate bonds to be formed and cleaved, respectively. Red solid lines in **6** correspond to red dotted lines in intermediate **D**.

Several molecules with different shapes are observed in the STM topography. Figure 2a shows a close-up view of a single molecule, having a mirror-symmetric structure, benzo[*mn*]benzo [8,9] phenanthro[3,4,5,6-*uvabc*]ovalene (**2**). To resolve the inner structure, the tip apex was terminated with a CO molecule [64,65]. The bond-resolved image shows the planar carbon skeleton in a rhombus shape, missing one hexagonal ring at the corner (Figure 2b). Possible reaction steps towards NG **2** from precursor **1** is displayed in Scheme 1. Dehydrocyclization of **1** would produce intermediate **A** with helical structure, which tends to strain-induced skeletal rearrangement [46,47]. Subsequent Diels-Alder cycloaddition [66] step afforded intermediate **B**. Then, NG **2** was obtained by direct ethyne extrusion.

Next, we investigate a product, which appears a slightly asymmetrical contrast in the STM topography, benzo[*mn*]benzo [8,9] phenanthro[3,4,5,6-*uvabc*]cyclopenta[*ef*]ovalene (**3**) (Figure 2d). The corresponding bond-resolved image shows the pentagonal ring fused at the zigzag edge as indicated by an arrow in Figure 2e. Thus, NG **3** could be formed through the skeletal rearrangement of intermediate **B** without the dissociation of ethyne (Figures 2f and Scheme 1). Similar reactions were also observed in our previous study [57]. Moreover, we also found another type of molecule in a fish-like shape, benzo[*mn*]benzo [8,9] phenanthro[3,4,5,6-*uvabc*]cyclopenta[*jk*]ovalene (**4**) (Figure 2g) whose pentagonal ring was located at a different site of the zigzag edge (Figure 2h) in comparison with NG **3**. The C_2_H_2_ group might migrate along the edge during the skeletal rearrangement of intermediate **B**, as indicated by a red arrow in Figure 2i. An asymmetric spindle-shape molecule, NG **5** (Figure 2j), having cyclopenta[*cd*]azulene moiety, was observed, as indicated by red arrows in Figure 2l. We assume that the seven-membered rings were formed through further cyclodehydrogenation of intermediate **A** to give intermediate **C**, which can be highly strained on surface due to the 2D confinement, inducing dissociation of ethyne to yield NG **5** (Scheme 1). We found the five-seven-five membered rings fused nanographene, NG **6** (Figure 2m), in which one 1,3-dimethylphenyl (C_8_H_9_) moiety at the right-hand side was cleaved (Figure 2n) during the dehydrocyclization of **1**. In comparison to **3** and **4**, the formation of the array of NG might suffer from more complex skeletal rearrangement, as displayed in Scheme 1. During these processes, the surface provides a catalytic and spatially confining environment that may influence the C – C bond reorganization and facilitate the overall rearrangements. These findings highlight the potential of skeletal rearrangement reactions to yield diverse NGs from designed precursor molecules. We also performed a statistical analysis of the products, which indicates a relatively uniform distribution, with molecules **4** and **5** occurring less frequently (Figure S5). Molecule **2** shows the highest selectivity among the observed products. In addition, several products (**6**, **7** and **8**), which involve fragment loss after C – C bond cleavage, also account for a significant fraction of the products. These results suggest that steric hindrance induced molecular rearrangements can efficiently incorporate nonbenzenoid rings into NGs.

**Figure 3.**
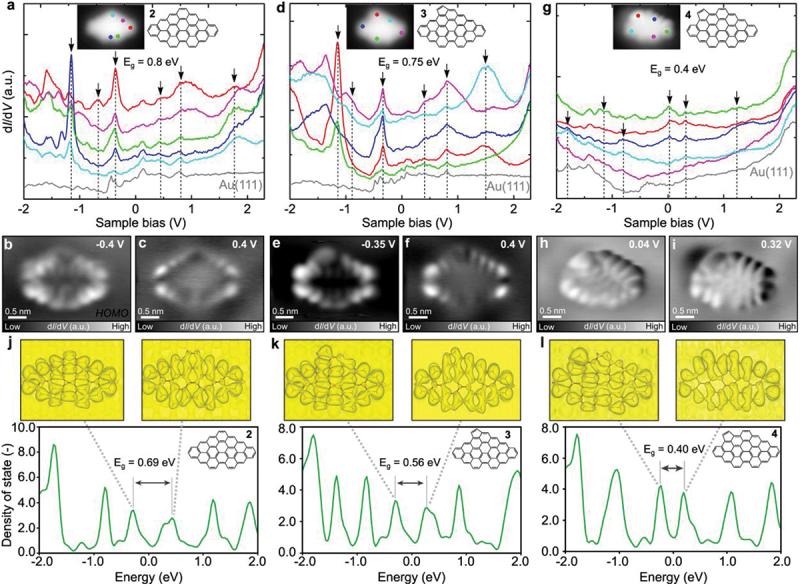
Electronic properties of **2**, **3** and **4**. (**a**) d*I*/d*V* spectra recorded at different sites over **2** (indicated by color dots in the inset) and the bare Au(111) surface. Constant current d*I*/d*V* maps measured at the sample bias voltages of (**b**) −0.4 V and (**c**) 0.4 V. (**d**) d*I*/d*V* spectra recorded at different sites over **3** (indicated by color dots in the inset) and the bare Au(111) surface. Constant current d*I*/d*V* maps measured at the bias voltages of (**e**) −0.35 V and (**f**) 0.4 V. (**g**) d*I*/d*V* spectra recorded at different sites over **4** (indicated by color dots in the inset) and the bare Au(111) surface. Constant current d*I*/d*V* maps measured at the sample bias voltages of (**h**) 0.04 V and (**i**) 0.32 V. (**j-l**) DFT calculated electronic density of states, projected onto the p-components of the carbon atoms for molecules **2**, **3** and **4** (lower panels), and the partial charge densities (upper panels) corresponding to the HOMOs and LUMOs, with the energy positions indicated by the dashed lines. Measurement parameters: *V* = 0.4 V, *I* = 100 pA, *V*_ac_ = 10 mV in (a), (d), (g).

The electronic properties of the NGs were investigated by STS measurements at 4.3 K. Figure 3a shows d*I*/d*V* curves recorded at specific sites over **2** (indicated by dots in different colors) and the bare Au(111) surface as a reference (grey curve). We found several peaks at −1.2 V, −0.6 V, −0.4 V, 0.4 V, 0.8 V and 1.8 V. The constant current d*I*/d*V* maps taken at the corresponding energies show the spatial distribution of electronic states (Figure 3b,c and Figure S6). The occupied state of −0.4 V distributes on the whole molecule, exhibiting stronger signals at the edges (Figure 3b), which is identified as the HOMO. The unoccupied state of 0.4 V (Figure 3c) exhibits nodal patterns at the edges, identified as the LUMO. Thus, **2** has a small HOMO-LUMO gap of 0.8 eV. It is worth noting that, despite the presence of the zigzag edges, no significant signal, relating to the magnetic properties, was detected near the Fermi level. We deduced that the closed shell character is caused by the close proximity of opposite spin configurations [67]. Next, we investigate **3**, which has an additional pentagonal ring. d*I*/d*V* curves taken above **3** also exhibit several peaks at −1.15 V, −0.8 V, −0.35 V, 0.4 V, 0.8 V and 1.5 V (Figure 3d). The d*I*/d*V* maps taken over **3** at the corresponding energies of −0.35 V and 0.4 V show similar features to those of **2**, while having a slight asymmetry, which can be assigned to HOMO and LUMO, respectively (Figure 3e,f). Thus, the HOMO-LUMO gap of **3** was 0.75 eV. The electronic states measured at other peaks (Figure S7) are localized at the pentagonal ring in **3**, which is consistent with findings from earlier theoretical [68,69] and experimental studies [70]. **4**, an isomer of **3**, has a pentagonal ring at a different zigzag edge site and exhibits a significantly smaller HOMO-LUMO gap of 0.4 eV (Figure 3g). The HOMO peak slightly exceeds the Fermi level, suggesting charge transfer between molecule **4** and the underlying substrate, potentially resulting in hole doping of molecule **4** [71]. Accordingly, Bader charge analysis based on the DFT-calculated charge densities shows that **4** carries a net charge of +0.328 e on Au(111). The corresponding d*I*/d*V* maps (Figure 3h,i, Figure S8) reveal that the electronic states of the HOMO and LUMO are delocalized across the entire molecule. This result reveals that the fused site of the pentagonal ring strongly affects the electrical properties of NGs. We were unable to investigate the electronic properties of **5** since the molecule accidentally diffused away from the tip on the surface during the STS measurement (Figure S9). The electronic property of **6** was also investigated (Figure S10). Figure 3j–l show the electronic density of states for the carbon p-component and the partial charge densities, corresponding to the HOMOs and LUMOs. Particularly, the calculated band gaps for **2** (0.69 eV) and **3** (0.56 eV), as well as the narrower gap of **4** (0.40 eV), are in good agreement with the experimentally measured results. We additionally performed simulated d*I*/d*V* maps (Figure S11). For **2** and **3**, the HOMO and LUMO maps match the experimental results with more edge nodes. For NG **4**, the maps show delocalized states across the molecule, also consistent with experiment. **3** and **4** are isomers, in which the pentagonal ring is fused to distinct positions of the zigzag edge. This small change leads to a relatively large variation in band gap, indicating that both the pentagonal ring and the zigzag edge are highly sensitive to electronic properties. Therefore, modifying the position of the pentagonal ring along the zigzag edge serves as an effective strategy for tuning the electronic characteristics.

**Figure 4.**
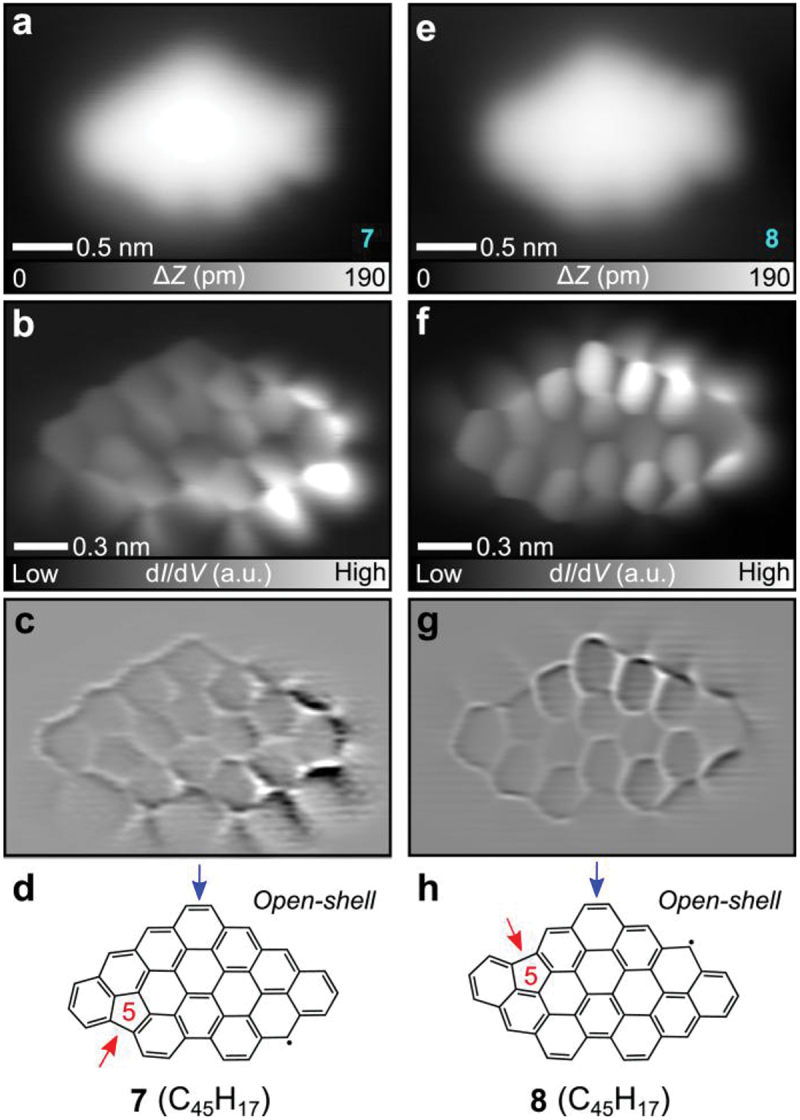
Synthesis of open-shell NGs. (a) Close-up STM topographies of **7**. (b) Bond resolved image of **7** and (c) the Laplace‐filtered image. (d) Chemical structure of **7**. (e) Close-up STM topographies of **8**. (f) Bond resolved image of **8** and (g) the Laplace‐filtered image. (h) Chemical structure of **8**. Measurement parameters: *V* = 200 mV and *I* = 5 pA in (a). *V* = 100 mV and *I* = 5 pA in (e). *V* = 1 mV, *V*_ac_ = 10 mV in (b, f).

Besides the above-investigated NGs, we found another type of molecule whose shape in the STM topography is at glance the same as that of **2**, named as **7** (Figure 4a). However, the bond resolved image shows stark contrast as the right-bottom of the molecule (Figure 4b), which indicates the presence of the spin-polarized state. We deduce that the product has one unpaired electron, leading to an open-shell structure. The corresponding Laplace-filtered image (Figure 4c) shows the formation of a pentagonal ring at the left-hand side, which was most probably formed by demethylation and subsequent dehydrocyclization [72,73]. Thus, the presence of the pentagonal ring leads to the S = 1/2 state (Figure 4d). The STM topography of another type of NG, named **8**, is almost identical to that of **7** (Figure 4e). However, the bond resolved image (Figure 4f) and Laplace-filtered image (Figure 4g) reveal that a pentagonal ring was embedded at a different site. Thus, **8** is a structural isomer of **7 (**Figure 4h), and both of these are most probably formed through the same type of the rearrangement as the formation of NG **2**. The bright feature at the upper-right of **8** indicates the open-shell character (Figure 4f). We also observed the singly occupied molecular orbitals (SOMOs), singly unoccupied molecular orbitals (SUMOs) and other electronic states of **7** and **8** (Figures S12, S13).

**Figure 5.**
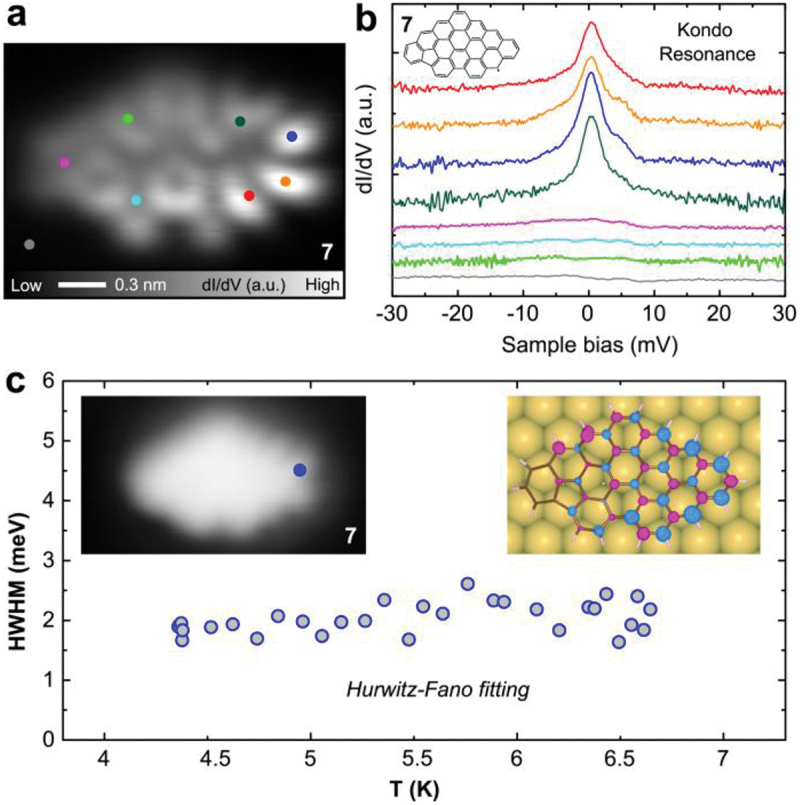
Magnetic properties of **7**. (a) Constant height d*I*/d*V* map of **7** measured at 0 V. (b) d*I*/d*V* spectra recorded at different sites as indicated by dots in (a). (c) Half width at half-maximum (HWHM) of Kondo resonance as the function of temperature, determined from Hurwitz-Fano lineshape fitting. Left inset: the blue dot on **7** indicates the measurement site. Right inset: DFT-calculated spin densities of **7**. Measurement parameters: *V* = 0 mV, *V*_ac_ = 1 mV in (a). *V* = 30 mV, *I* = 200 pA, *V*_ac_ = 0.3 mV in (b).

To investigate the magnetic properties of **7**, we conducted STS measurements near the Fermi level at several sites, as indicated by dots in Figure 5a. d*I*/d*V* spectra have distinct peaks at 0 V only on the right-hand side of the molecule (Figure 5b). The zero-bias peak can be attributed to the Kondo resonance, in which the net spin of the molecule is screened by conduction electrons of the underlying Au(111) substrate. Therefore, **7** has an S = 1/2 character. To resolve the spatial distribution of spin-polarized state, the constant-height d*I*/d*V* map was taken at 0 V (Figure 5a). We found that the d*I*/d*V* signal was greatest in the bottom right region and became weaker with increasing distance from the area. Thus, the spin state is localized at the zigzag edge. To gain a deeper insight into the Kondo resonance, we performed the temperature-dependent STS measurement at the site of **7** (left inset of Figure 5c). The spectra were analyzed using the recently reported Hurwitz-Fano lineshape model [74], which accounts for multiple factors including tip effect and temperature, resulting into relatively consistent half width at half-maximum (HWHM) values shown in Figure 5c. The Kondo temperature (*T*_K_) of **7** on Au(111) was estimated to be ~ 6 K using HWHM = 3.92 *k*_B_*T*_K_. The DFT-calculated spin densities of **7** show that both the spin densities are more localized on the zigzag edges at right-hand sides (right inset of Figure 5c), which agree well with the d*I*/d*V* map. To investigate the influence of the zigzag edge on the left-hand side, their extended derivatives of **7** (**9** and **10**) were analyzed (Figure S14). We found that the net spin polarization and the electron transfer increase to 0.956 µ_B_ and 0.379 e (**9**) or 0.963 µ_B_ and 0.412 e (**10**) from 0.900 µ_B_ and 0.367 e (**7**) with the molecule size. Furthermore, the spin density at the zigzag edge of the left-hand side becomes greater while the one with the pentagonal ring dismisses the intensity. In addition, molecule **8** also exhibits spin-polarized characteristics on Au(111) (Figure S15).

## 4. Conclusions

We have fabricated seven types of NGs through cyclodehydrogenation of an anthryltetraphene-based molecule, involving skeletal rearrangement on Au(111). The rearrangement is triggered by the spatial overlap of carbon atoms in the three-dimensional configuration. Six of the obtained NGs highlighted the nonbenzenoid structures with pentagonal and heptagonal rings. Their inner structures were characterized by bond-resolved STM. Their electronic and magnetic properties were further investigated by STS measurements. Among them, five types of closed-shell NGs exhibit rich varieties of electronic structures with different HOMO-LUMO gaps, particularly the combination of pentagonal rings and zigzag edges, which can significantly modify the electronic properties. The open-shell NGs with a pentagonal ring in the zigzag edge exhibit delocalized zero-bias states in the d*I*/d*V* spectra, identified as Kondo resonances on the metal surface. The relationship between the NG size and the magnetic property was identified with the density functional theory calculation. This finding would be beneficial to construct more intriguing nonbenzenoid carbon nanostructures by molecular rearrangement.

## Supplementary Material

Supplemental Material
