# Supplementary material for: On-surface synthesis of nonbenzenoid nanographenes through skeletal rearrangement reactions on Au(111)
Source: Sci Technol Adv Mater. 2026 Jan 23;27(1):2619342. doi: 10.1080/14686996.2026.2619342 (PMC12903936; doi:10.1080/14686996.2026.2619342)
Supplement: Supplemental Material [file TSTA_A_2619342_SM0894.docx]

Supplementary Materials for

On-surface synthesis of nonbenzenoid nanographenes through skeletal rearrangement reactions on Au(111)

Kewei Sun^1,2^, Xiushang Xu^3^, Atsushi Ishikawa^4^*, Akimitsu Narita^3^*, Shigeki Kawai^2,5^*

^1^*International Center for Young Scientists, National Institute for Materials Science, 1-2-1 Sengen, Tsukuba, Ibaraki 305-0047, Japan.*

^2^*Center for Basic Research on Materials, National Institute for Materials Science, 1-2-1 Sengen, Tsukuba, Ibaraki 305-0044, Japan.*

^3^*Organic and Carbon Nanomaterials Unit, Okinawa Institute of Science and Technology Graduate University, 1919-1 Tancha, Onna-son, Kunigami-gun, Okinawa 904-0495, Japan.*

^4^*Department of Transdisciplinary Science and Engineering, School of Environment and Society, Institute of Science Tokyo, 2-12-1 Ookayama, Meguro-ku, Tokyo, 152-8552, Japan.*

^5^*Graduate School of Pure and Applied Sciences, University of Tsukuba, Tsukuba 305-8571, Japan.*


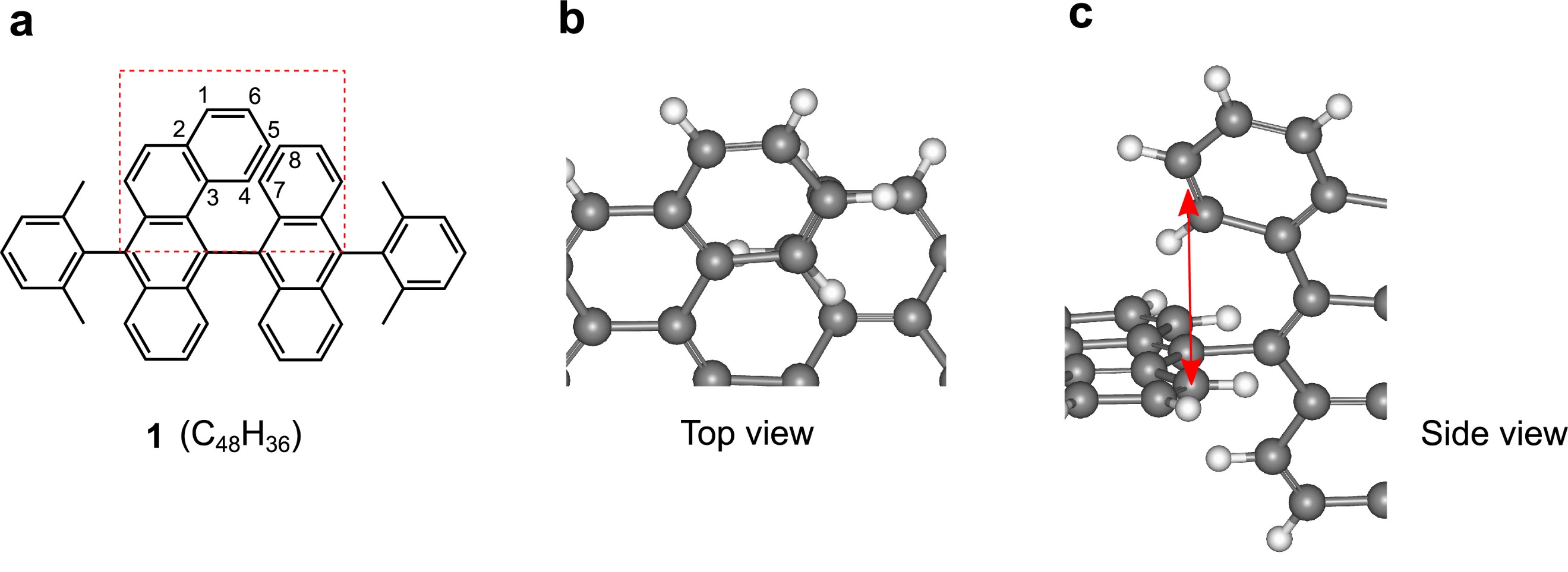


**Figure S1**. Molecule **1**. (a) Chemical structure of precursor molecule **1**. (b) Top view of the region highlighted by a rectangle in (a). (c) Corresponding side view.

**
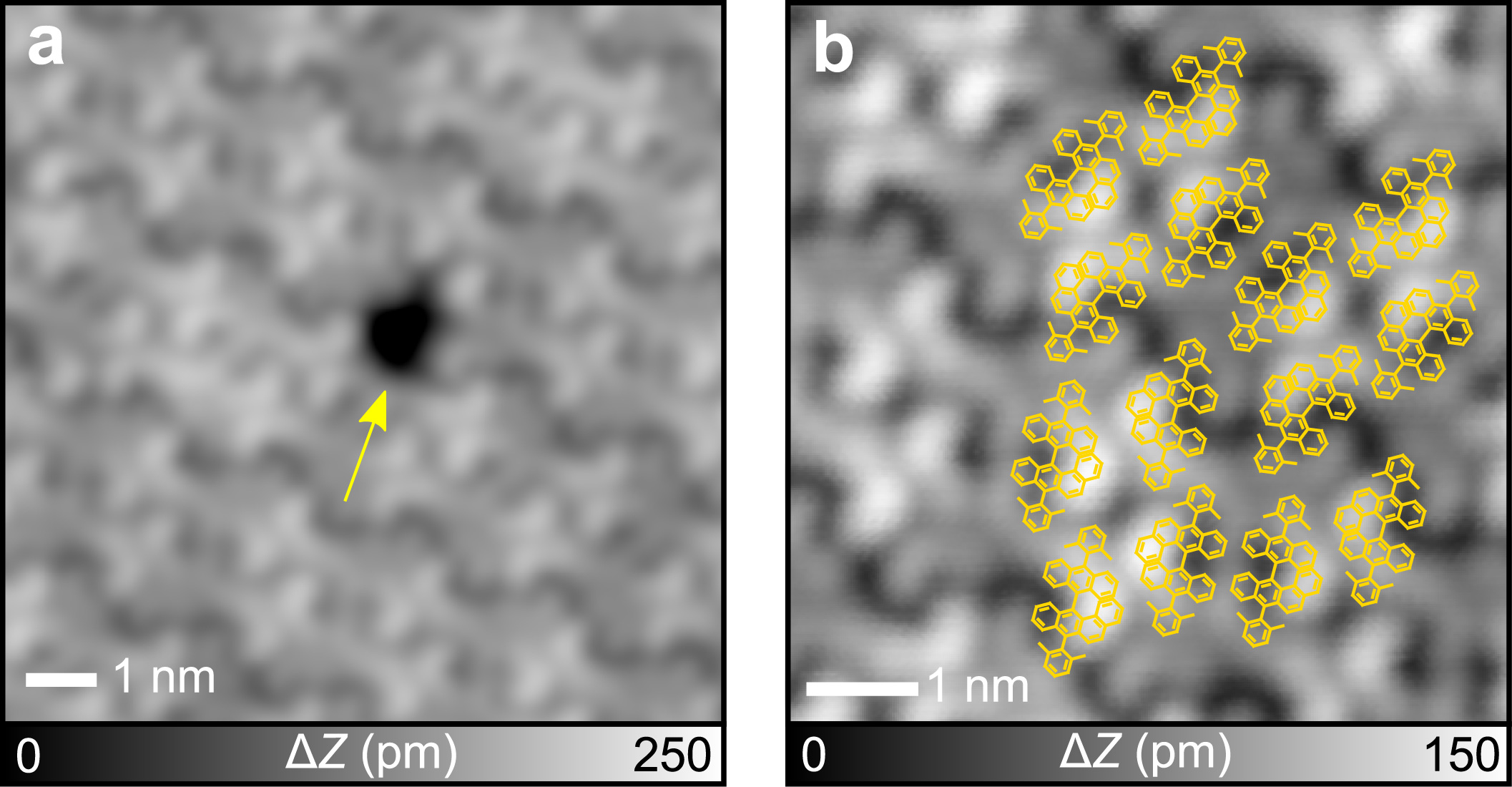
**

**Figure S2**. Self-assembled **1** on Au(111). (a) Scanning tunneling microscopy (STM) topography of the self-assembled structures. The black site (indicated by the arrow) represents the area after removing one molecule via tip manipulation. Whereby we can determine the arrangement of molecules. (b) STM topography of self-assembled molecules. The chemical structures of **1** are superimposed. Measurement parameters: *V* = 200 mV and *I* = 5 pA in (a). *V* = 200 mV and *I* = 10 pA in (b).


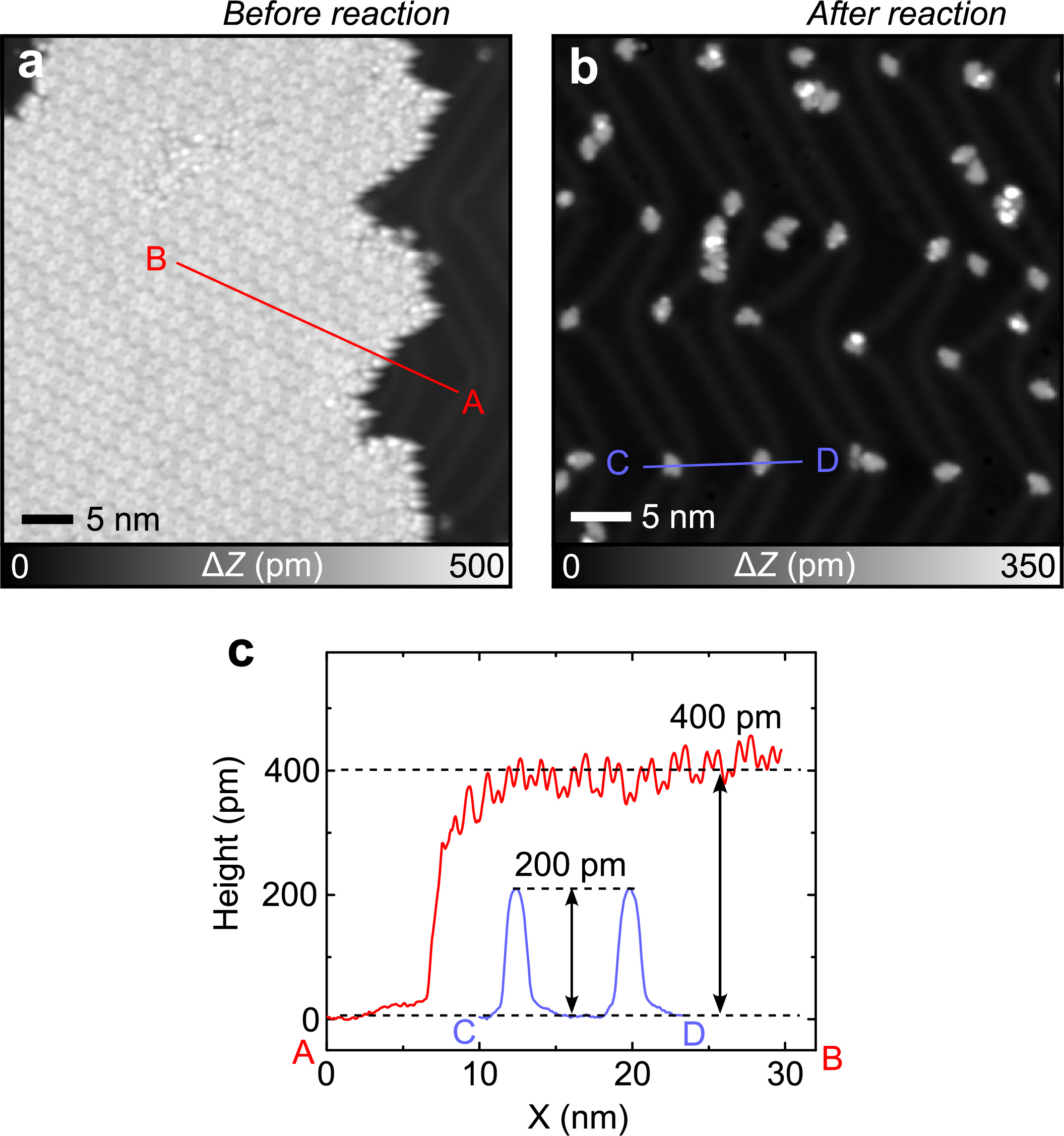


**Figure S3**. Comparison of molecular heights before and after reaction. (a,b) STM topographies before (a) and after (b) reaction. (c) Line profiles taken along the lines indicated in (a) and (b). Measurement parameters: *V* = 200 mV and *I* = 2 pA.


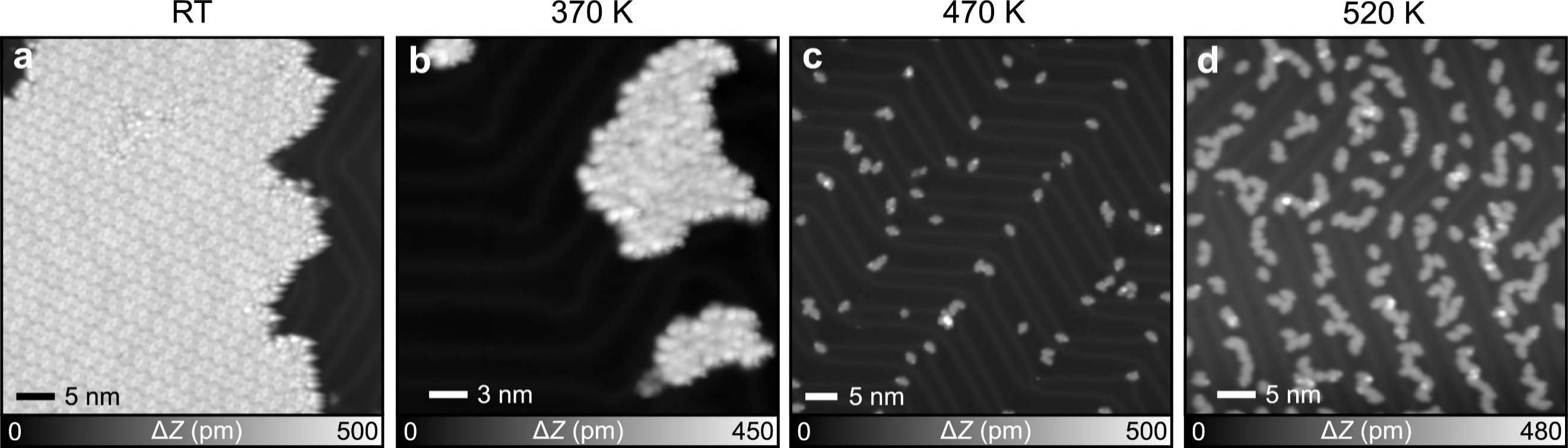


**Figure S4**. Temperature-dependent STM images of molecules on Au(111). (a) STM topography of as-deposited molecules **1** on Au(111). (b-d) STM topographies of Au(111) surfaces after heating to (b) 370 K, (c) 470 K and (d) 520 K. Measurement parameters: *V* = 200 mV and *I* = 2 pA in (a, c). *V* = 200 mV and *I* = 10 pA in (b, d).


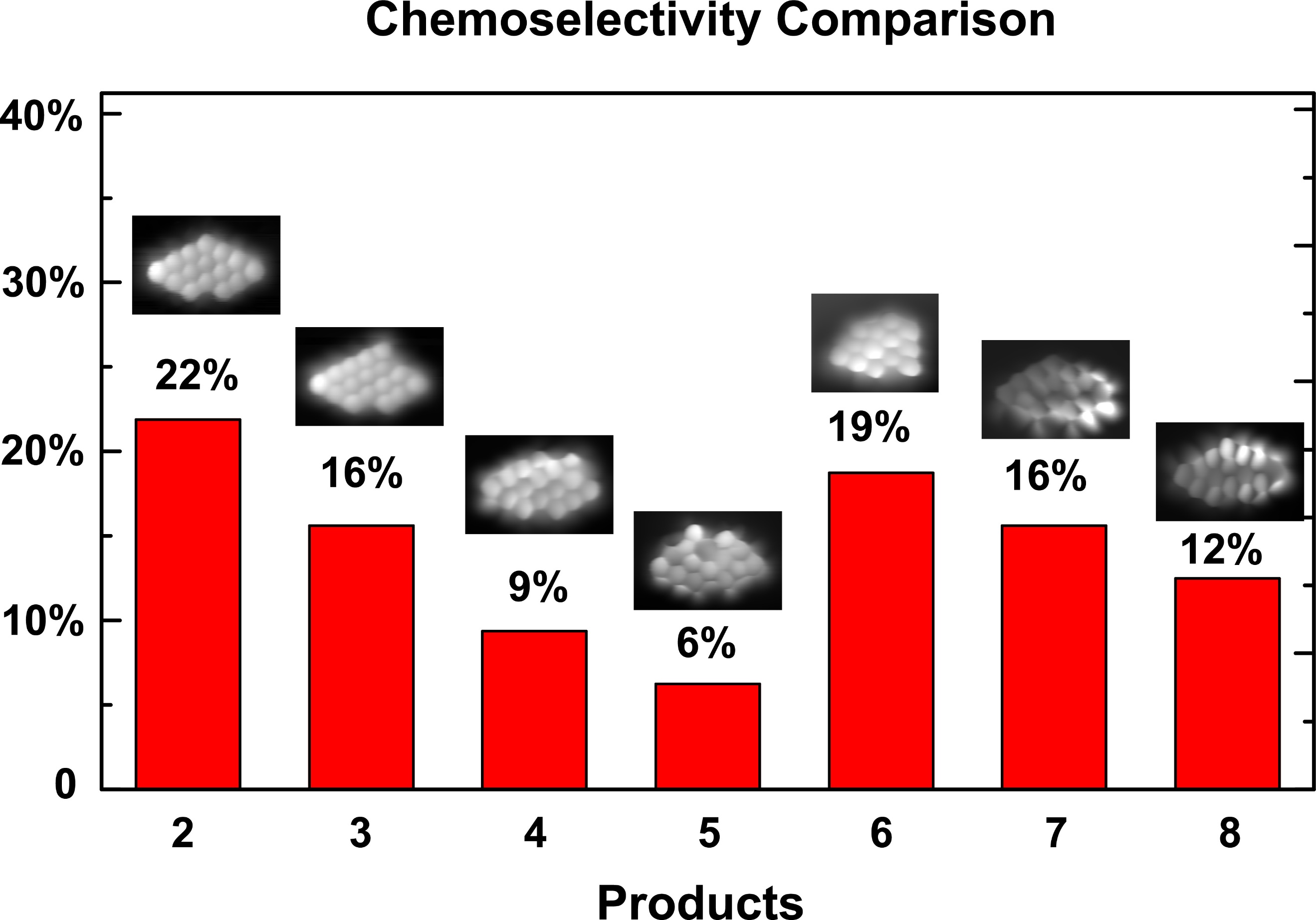


**Figure S5**. Statistical analysis of the chemoselectivity among seven product types resulting from the rearrangement reactions of molecule **1** on Au(111). Here, structures that were difficult to identify, as well as already fused oligomers, were not included in this analysis.


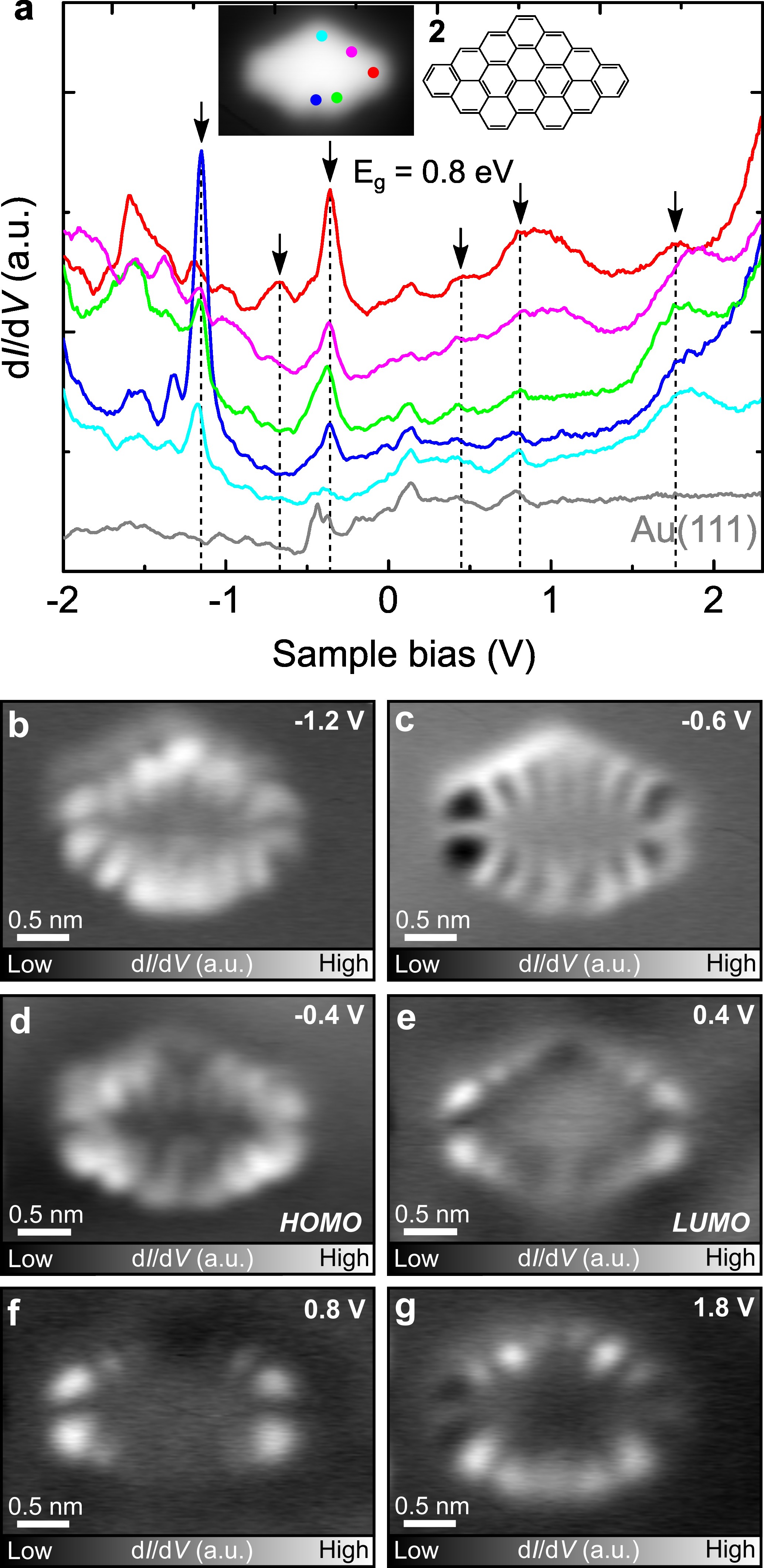


**Figure S6**. Electronic properties of NG **2**. (a) d*I*/d*V* spectra recorded at different sites above over individual **2** (indicated by color dots in the inset image) and the bare Au(111) surface. (b-g) A series of constant current d*I*/d*V* maps measured at different sample bias voltages: (b) −1.2 V, (c) −0.6 V, (d) −0.4 V, (e) 0.4 V, (f) 0.8 V and (g) 1.8 V. Measurement parameters: *V* = 0.4 V, *I* = 100 pA, *V*_ac_ = 10 mV in (a).


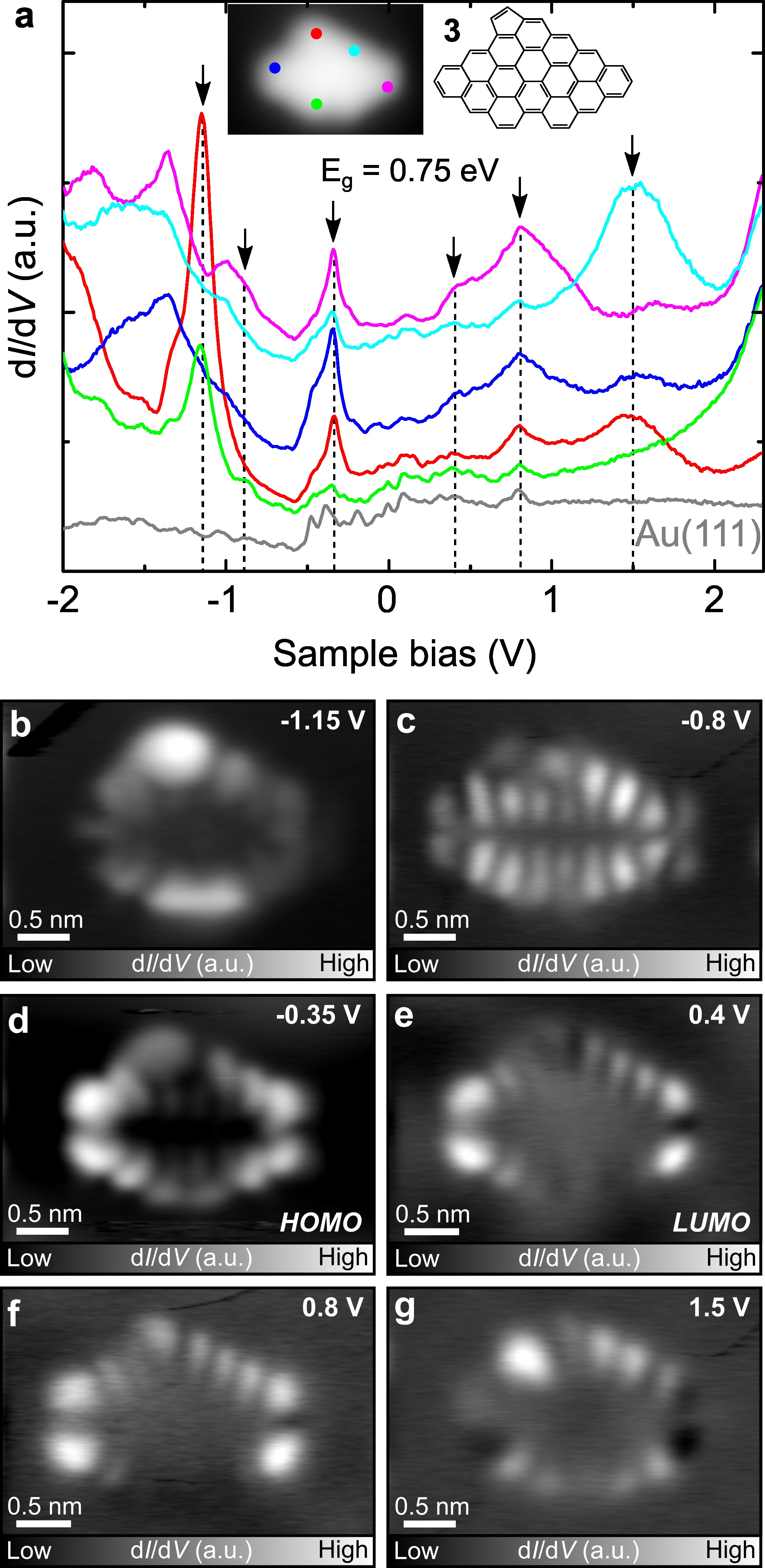


**Figure S7**. Electronic properties of NG **3**. (a) d*I*/d*V* spectra recorded at different sites above over individual **3** (indicated by color dots in the inset image) and the bare Au(111) surface. (b-g) A series of constant current d*I*/d*V* maps measured at different sample bias voltages: (b) −1.15 V, (c) −0.8 V, (d) −0.4 V, (e) 0.4 V, (f) 0.8 V and (g) 1.5 V. Measurement parameters: *V* = 0.4 V, *I* = 100 pA, *V*_ac_ = 10 mV in (a).


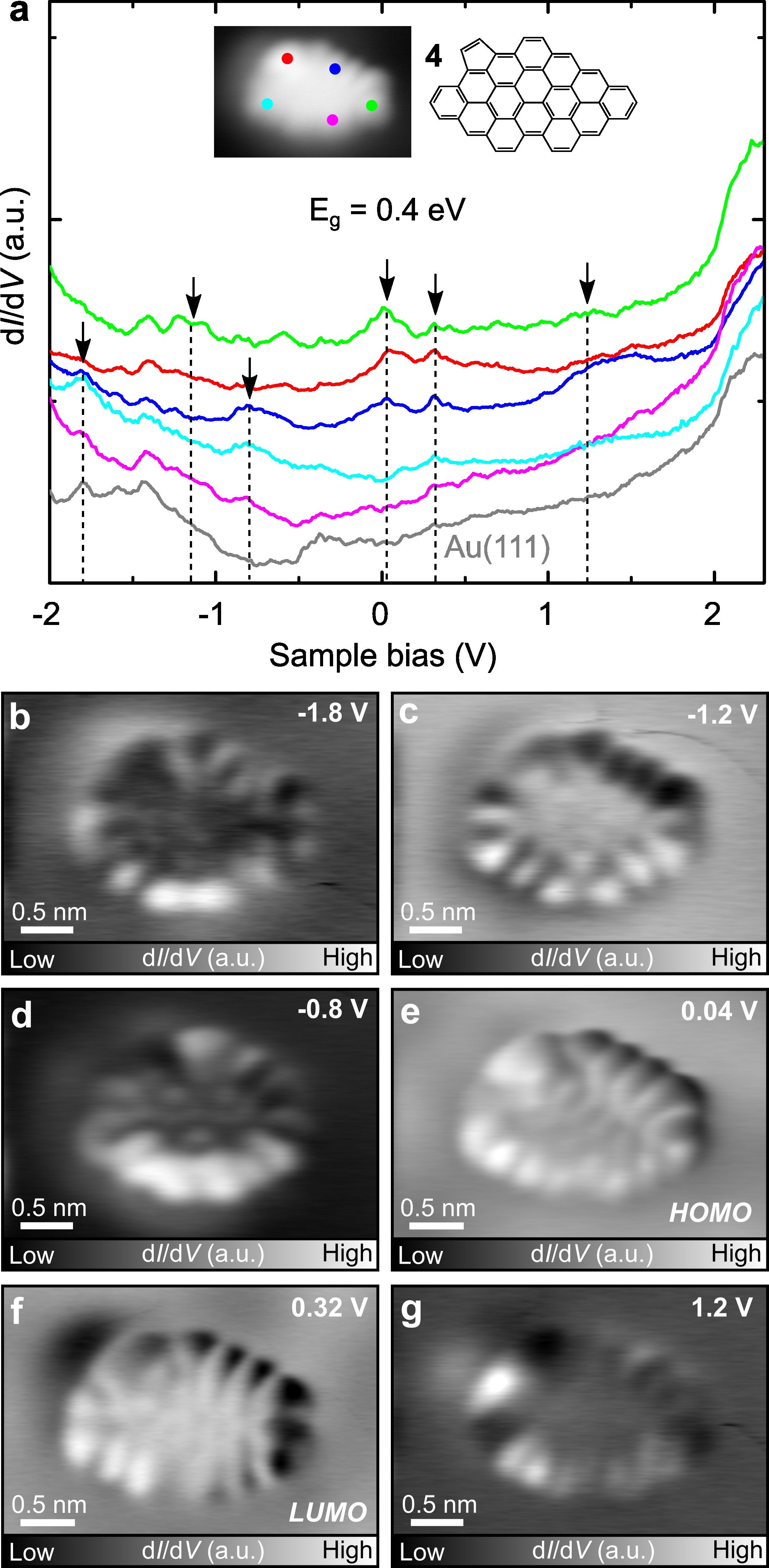


**Figure S8**. Electronic properties of NG **4**. (a) d*I*/d*V* spectra recorded at different sites above over individual **4** (indicated by color dots in the inset image) and the bare Au(111) surface. (b-g) A series of constant current d*I*/d*V* maps measured at different sample bias voltages: (b) −1.8 V, (c) −1.2 V, (d) −0.8 V, (e) 0.04 V, (f) 0.32 V and (g) 1.2 V. Measurement parameters: *V* = 0.4 V, *I* = 100 pA, *V*_ac_ = 10 mV in (a).


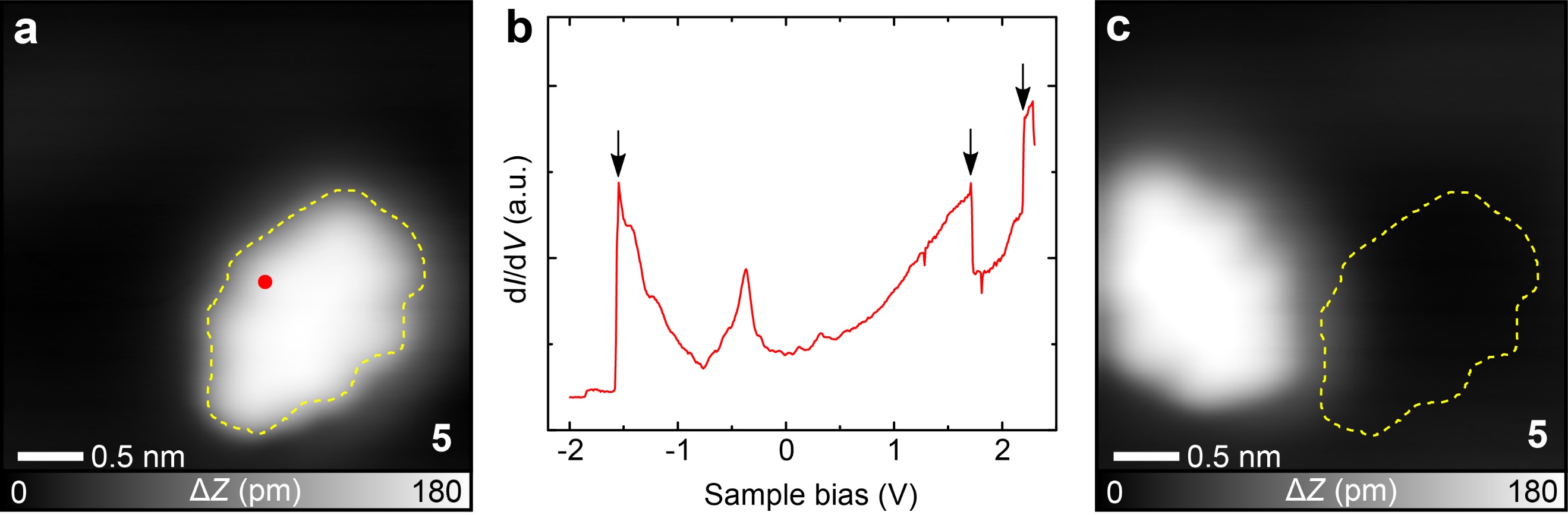


**Figure S9**. STS measurement on NG **5**. (a) STM topography of individual **5** on Au(111). (b) d*I*/d*V* spectrum taken at the site indicated by red dot in (a). The abrupt changes in the curve (marked by arrows) indicate the movement of **5**. (c) STM topography of individual **5** after STS measurement. **5** has moved. The yellow dashed frame indicates the original positions of **5**. Measurement parameters: *V* = 200 mV, *I* = 5 pA in (a), (c).


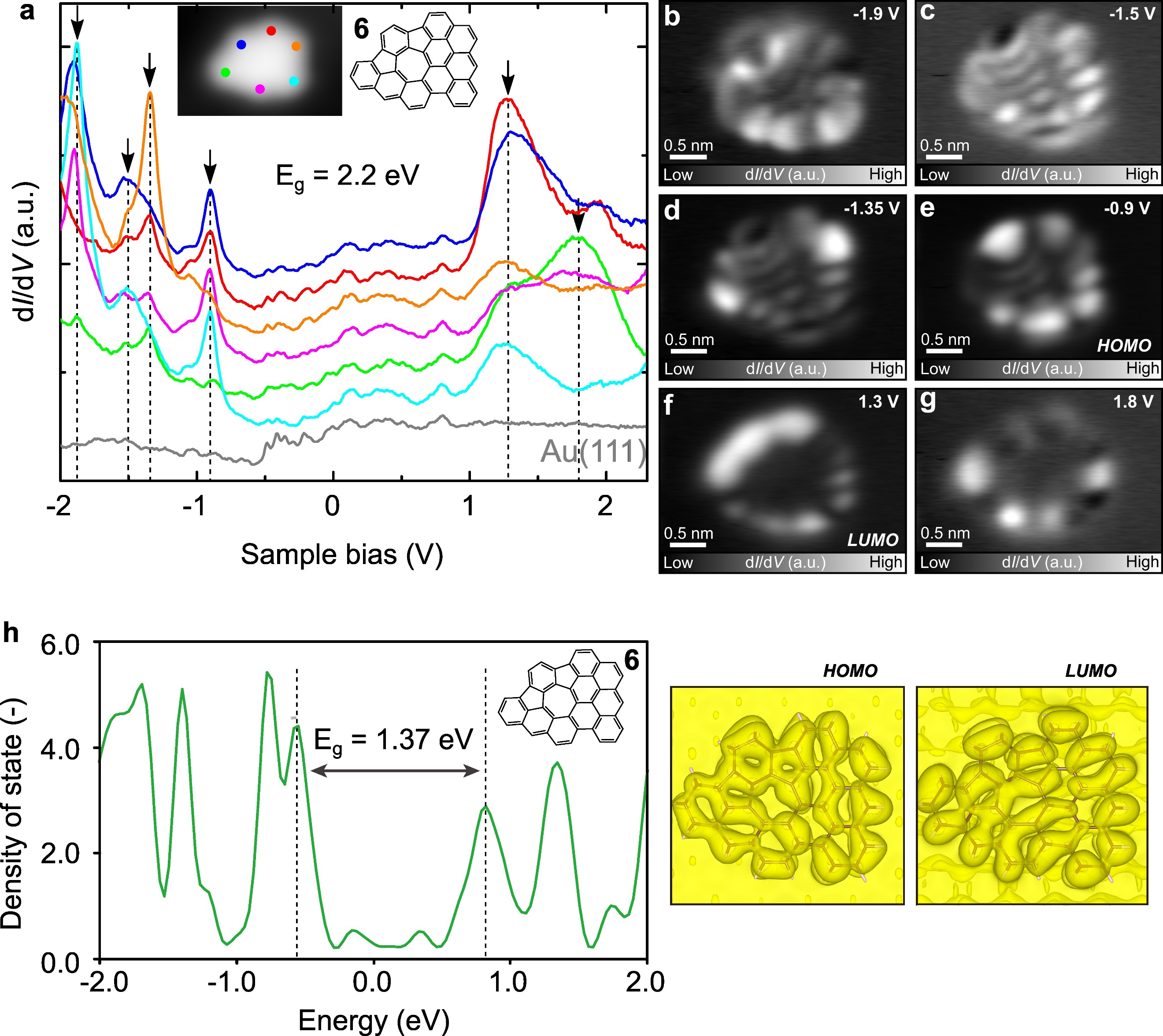


**Figure S10**. Electronic properties of NG **6**. (a) d*I*/d*V* spectra recorded at different sites above over individual **6** (indicated by color dots in the inset image) and the bare Au(111) surface. (b-g) A series of constant current d*I*/d*V* maps measured at different sample bias voltages: (b) −1.9 V, (c) −1.5 V, (d) −1.35 V, (e) −0.9 V, (f) 1.3 V and (g) 1.8 V. (h) Density functional theory (DFT)-calculated electronic density of states, projected onto the p-components of the carbon atoms for NG **6** (left panel), along with the partial charge densities (right panel) corresponding to the highest occupied molecular orbitals (HOMOs) and lowest unoccupied molecular orbitals (LUMOs). Measurement parameters: *V* = 0.4 V, *I* = 100 pA, *V*_ac_ = 10 mV in (a).


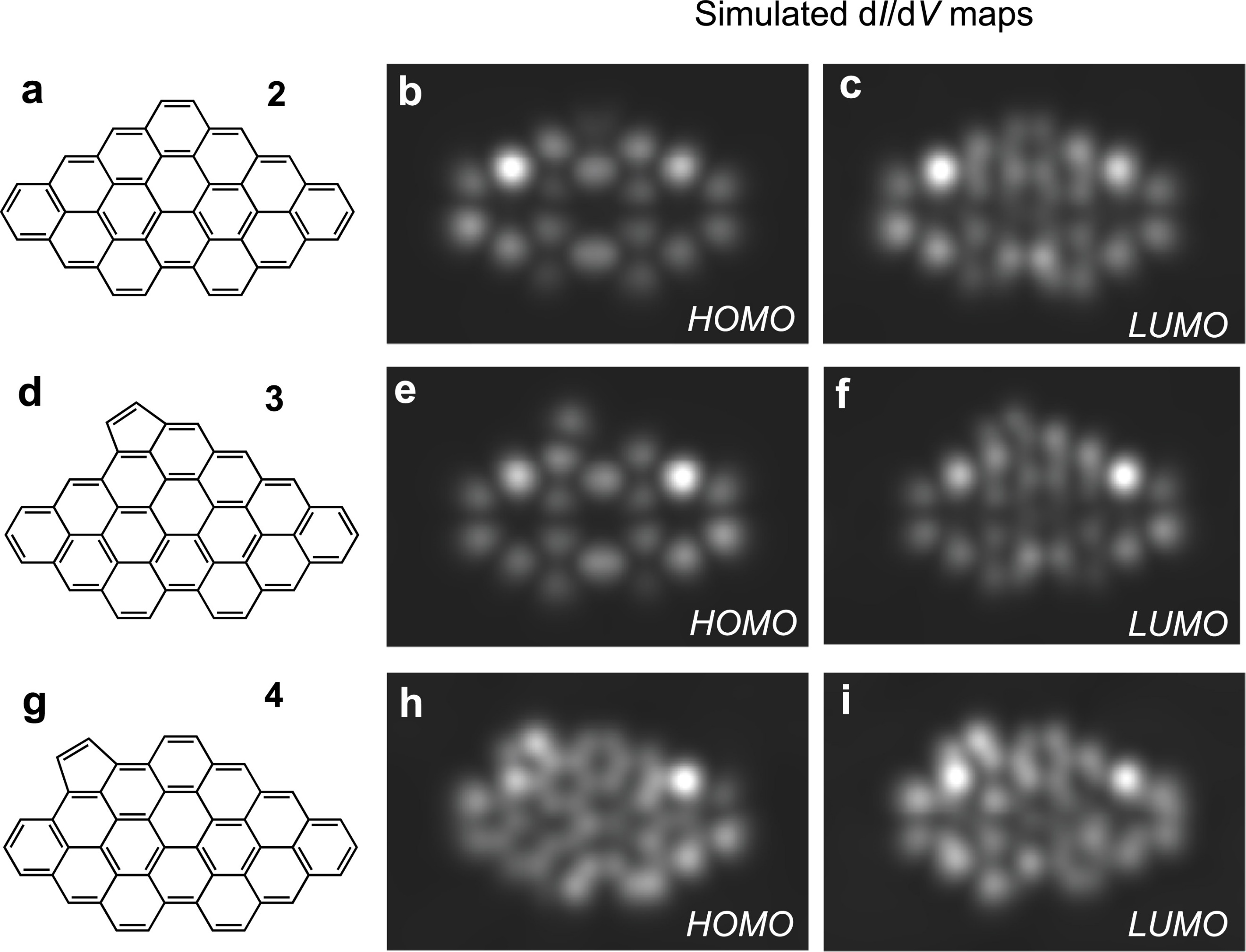


**Figure S11**. Simulated d*I*/d*V* maps of nano graphenes (NG)s **2**,**3**,**4**. (a, d, g) Chemical structures of **2**, **3**, and **4**. (b, c, e, f, h, i) DFT-simulated d*I*/d*V* maps of the HOMO and LUMO states, obtained from the DFT-calculated local density of states (LDOS) using the Tersoff–Hamann approximation. The energies were set to −0.3 eV (HOMO) and 0.3 eV (LUMO) for **2** and **3**, and −0.1 eV (HOMO) and 0.1 eV (LUMO) for **4**. The maps were generated in constant-height mode using the py4vasp package, with the tip positioned 1.0 Å above the surface.


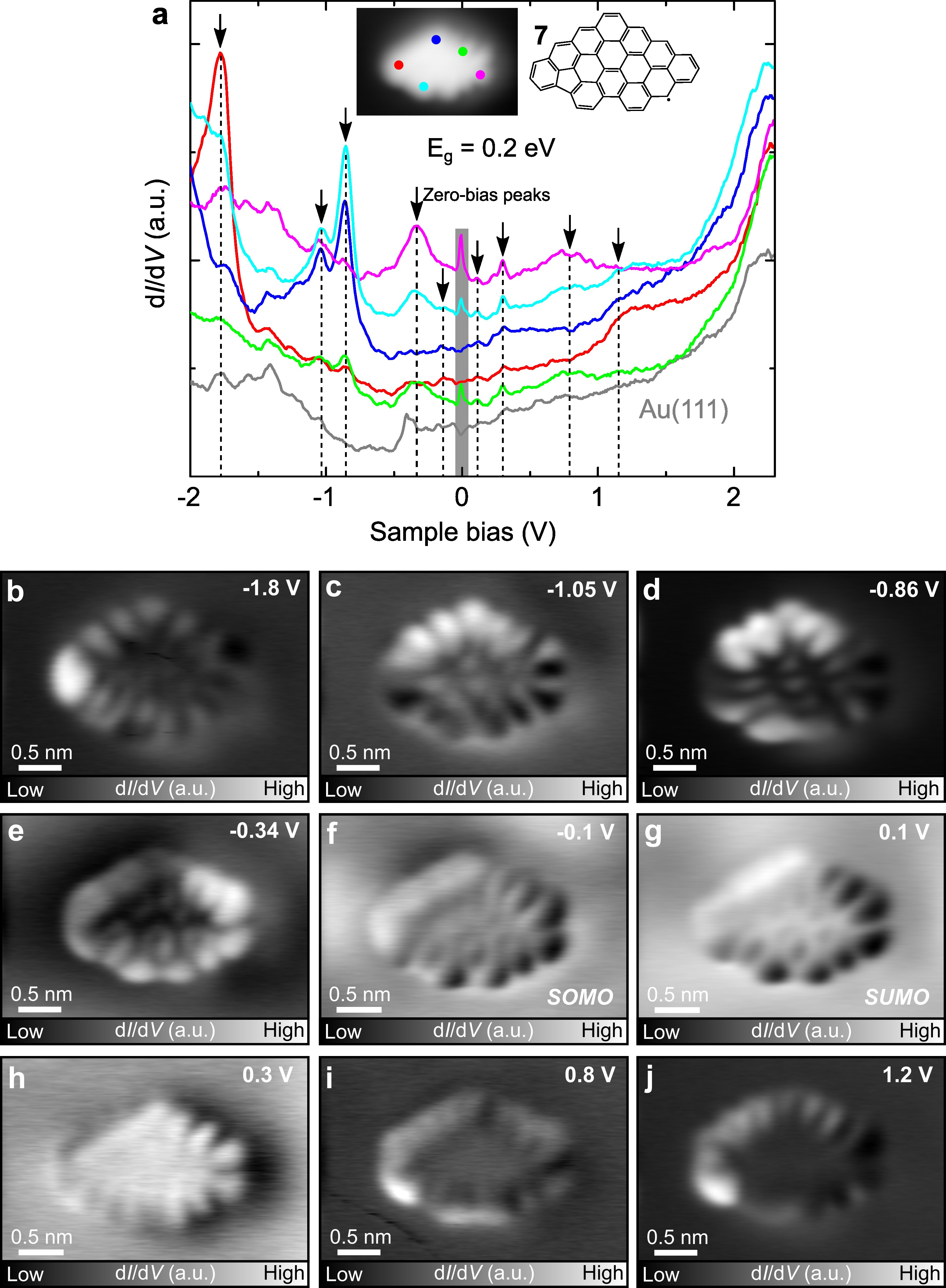


**Figure S12**. Electronic properties of NG **7**. (a) d*I*/d*V* spectra recorded at different sites above over individual **7** (indicated by color dots in the inset image) and the bare Au(111) surface. (b-j) A series of constant current d*I*/d*V* maps measured at different sample bias voltages: (b) −1.8 V, (c) −1.05 V, (d) −0.86 V, (e) −0.34 V, (f) −0.1 V, (g) 0.1 V, (h) 0.3 V, (i) 0.8 V and (j) 1.2 V. Measurement parameters: *V* = 0.4 V, *I* = 100 pA, *V*_ac_ = 10 mV in (a).


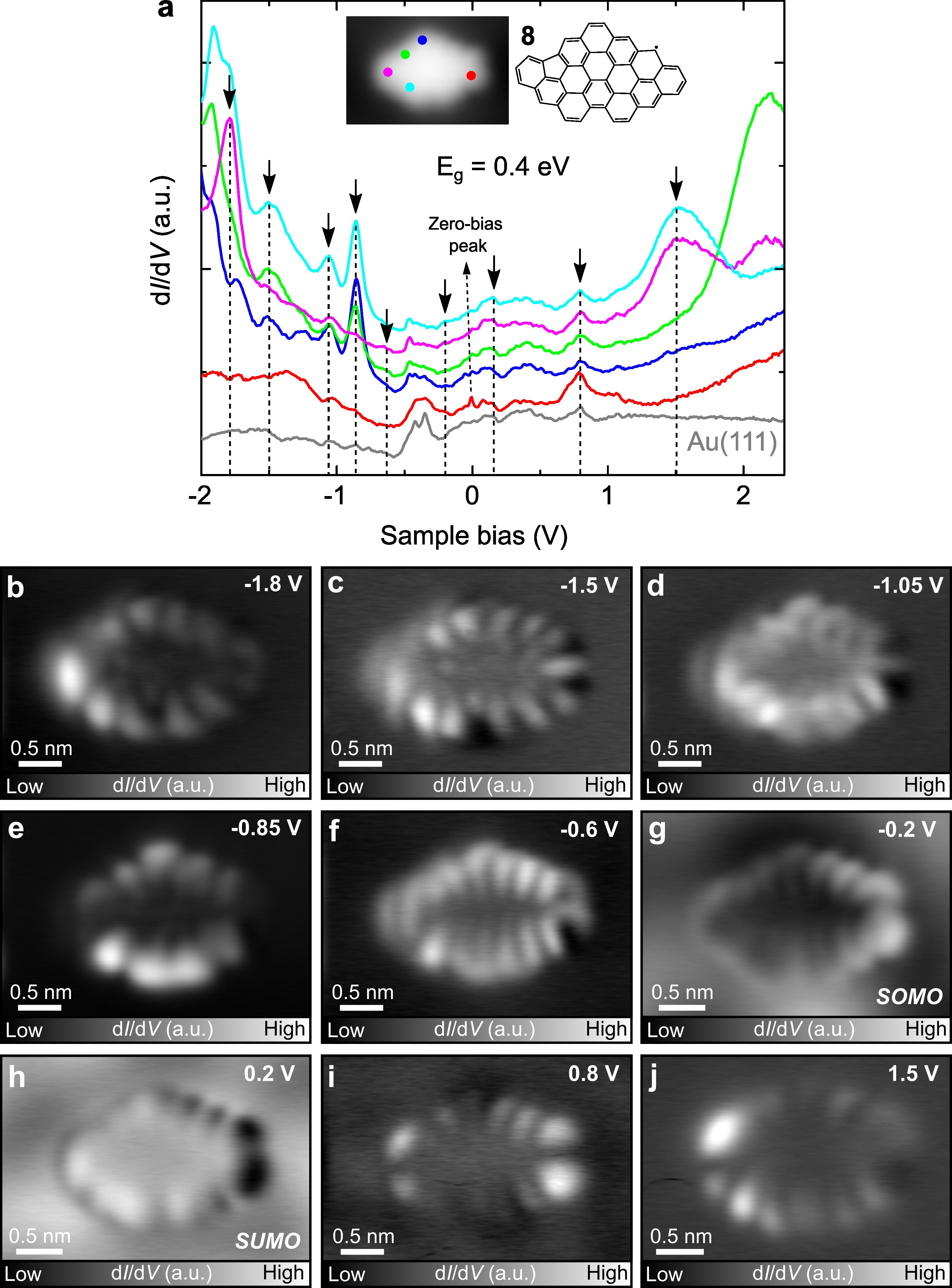


**Figure S13**. Electronic properties of NG **8**. (a) d*I*/d*V* spectra recorded at different sites above over individual **8** (indicated by color dots in the inset image) and the bare Au(111) surface. (b-j) A series of constant current d*I*/d*V* maps measured at different sample bias voltages: (b) −1.8 V, (c) −1.5 V, (d) −1.05 V, (e) −0.85 V, (f) −0.6 V, (g) −0.2 V, (h) 0.2 V, (i) 0.8 V and (j) 1.5 V. Measurement parameters: *V* = 0.4 V, *I* = 100 pA, *V*_ac_ = 10 mV in (a).


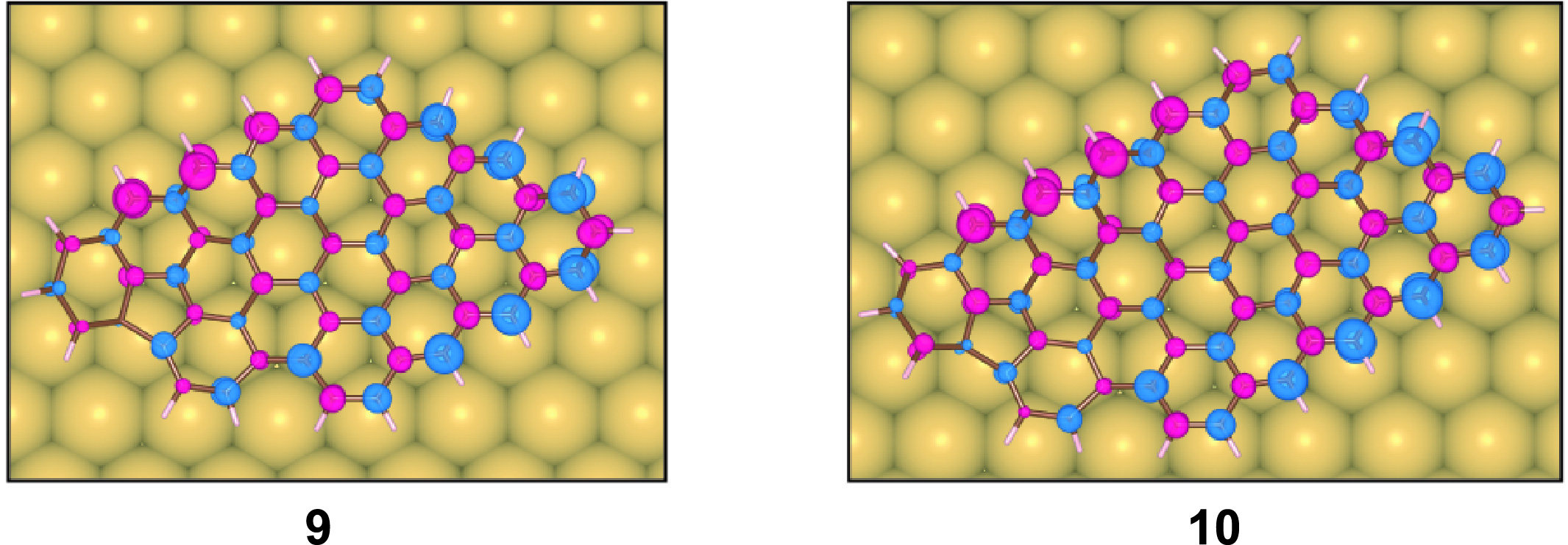


**Figure S14**. DFT-calculated spin densities of extended derivatives of **7** (**9** and **10**).

Zero-energy peaks were also observed in the d*I*/d*V* spectra recorded at the sites above molecule **8** (Figure S15a). The spatial distribution of the spin-polarized state (inset of Figure S15a) shows a stripe-like pattern slightly different from that of molecule **7** (Figure 5b). The calculated spin density of **8** (Figure S15b) is similar to that of **7,** with the spin mainly localized at the zigzag edge on the right-hand side. Bader charge analysis further shows that both **7** and **8** are positively charged due to electron transfer to the gold substrate, with net charges of 0.367 e and 0.376 e, respectively.


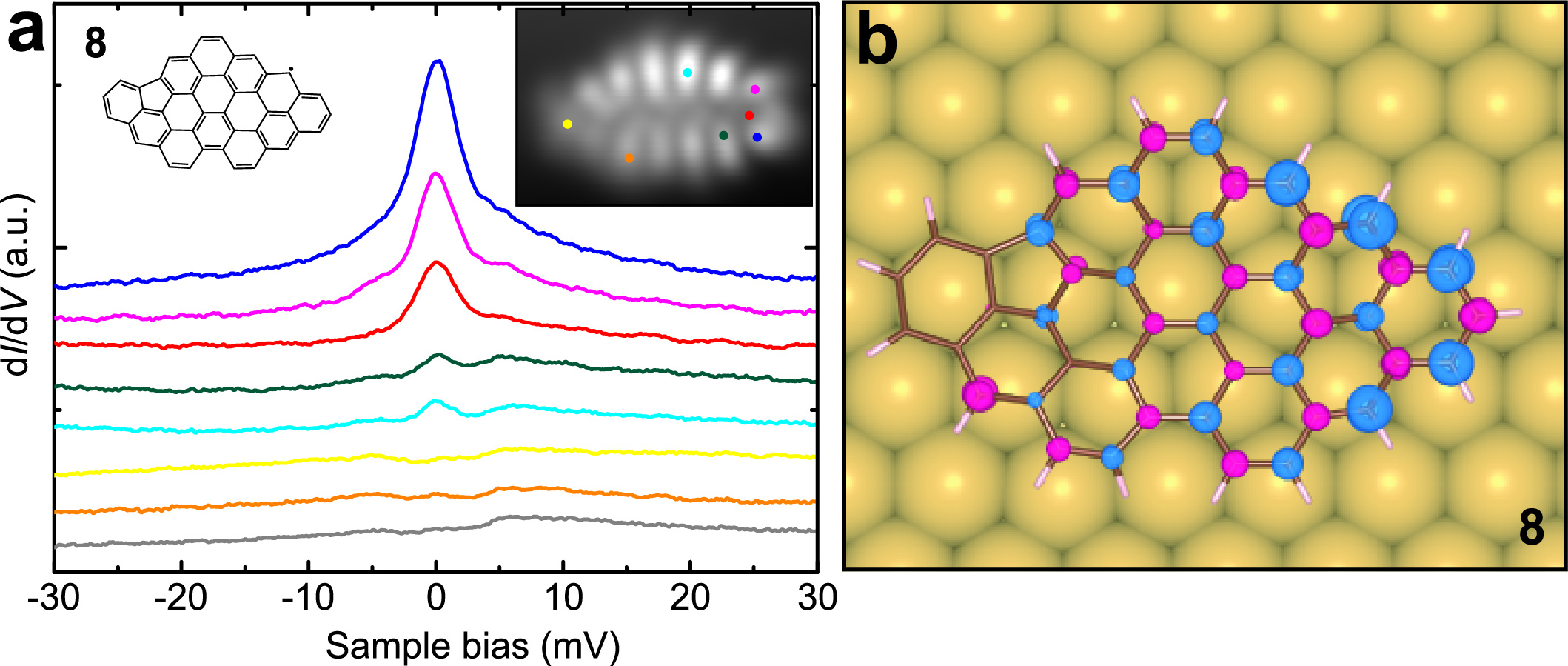


**Figure S15**. Magnetic properties of **8**. (a) d*I*/d*V* spectra recorded at different sites, as indicated by the colored dots in the inset. (b) DFT-calculated spin densities of **8**. Measurement parameters: *V* = 0.3 V, *I* = 200 pA, *V*_ac_ = 1 mV in (a).

**Figure S16.** Synthetic scheme for 7-(2,6-dimethylphenyl)-12-(10-(2,6-dimethylphenyl)anthracen-9-yl)tetraphene (**1**).

7-(2,6-dimethylphenyl)-12-[10-(2,6-dimethylphenyl)anthracen-9-yl]tetraphene (**1**)

A 30-mL Schlenk tube was charged with compound **9** (60 mg, 0.11 mmol), 2,6-dimethylphenylboronic acid (48 mg, 0.33 mmol), Pd_2_(dba)_3_ (10 mg, 11 μmol), SPhos (9.0 mg, 22 μmol), K_3_PO_4_ (140 mg, 0.660 mmol), and anhydrous toluene (5 mL) under argon atmosphere. The reaction mixture was subjected to freeze-pump-thaw cycles (3 times) and heated at 96 °C for 20 h under argon atmosphere. After cooling to room temperature, the resulting mixture was poured into water (50 mL), and the aqueous layer was extracted with CH_2_Cl_2_ (30 mL) for three times. The separated organic phases were combined, washed with brine, dried over MgSO_4_, and evaporated. The residue was purified by silica gel column chromatography (eluent: hexane: CH_2_Cl_2_ = 10:1) to give the title compound as yellow solid (43 mg, 65% yield). ^1^H NMR (500 MHz, CDCl_3_) *δ* 7.68 – 7.60 (m, 4H), 7.53 – 7.43 (m, 5H), 7.43 – 7.38 (m, 4H), 7.35 – 7.30 (m, 4H), 7.25 – 7.21 (m, 3H), 7.16 – 7.11 (m, 2H), 6.87 (d, *J* = 8.7 Hz, 1H), 6.55 (t, *J* = 7.9 Hz, 1H), 2.12 (s, 3H), 2.06 (s, 3H), 2.03 (s, 6H).^13^C NMR (126 MHz, CDCl_3_) *δ* 138.58, 137.91, 137.81, 137.76, 137.65, 137.02, 136.66, 136.20, 133.35, 133.08, 132.82, 131.02, 130.91, 129.98, 129.93, 129.36, 128.98, 128.47, 128.19, 127.86, 127.82, 127.68, 127.63, 126.78, 126.30, 126.15, 126.08, 126.00, 125.71, 125.59, 125.35, 31.62, 22.69, 20.39, 20.10, 14.17. HRMS (MALDI-TOF, Positive): *m/z* Calcd For C_48_H_36_^+^: 612.2812 [M]^+^, found: 612.2823.

**Figure S17**. ^1^H NMR spectrum of compound **1** in CDCl_3_ (500 MHz, 298 K).

**Figure S18**. ^13^C NMR spectrum of compound **1** in CDCl_3_ (125 MHz, 298 K).
